# How Follow-Up Neuroimaging Techniques Enhance Care for a Veteran with Combat-Related TBI and PTSD

**DOI:** 10.1192/j.eurpsy.2025.1666

**Published:** 2025-08-26

**Authors:** O. Inanc, D. Erbek, B. Garip

**Affiliations:** 1 Gulhane Training and Research Hospital, Ankara, Türkiye; 2 Psychiatry, Gulhane Training and Research Hospital, Ankara, Türkiye

## Abstract

**Introduction:**

As traumatic brain injury (TBI) is a significant concern among military veterans, ongoing neuroimaging is a beneficial tool for monitoring functional brain changes and evaluating the progression of symptoms.

**Objectives:**

Highlighting the importance of follow-up neuroimaging assessments in guiding treatment adjustments and understanding the evolving relationship between TBI, post-traumatic stress disorder (PTSD), and neurocognitive dysfunction.

**Methods:**

A 27-year-old male veteran injured by an IED experienced trauma to the right side of his body, resulting in 80% vision loss in the right eye and 20% in the left. He reported memory gaps and sleep disturbances. After inpatient and outpatient rehabilitation, he was prescribed Olanzapine (5 mg/day), Quetiapine (150 mg/day), and Venlafaxine (75 mg/day). On his second admission for increased sleep disturbances and anxiety, Quetiapine was increased to 200 mg/day. One year later, the patient developed new cognitive impairments and reported memory deficits and anterograde amnesia, concurrently PET scans revealed hypometabolism in the frontal lobe.

**Results:**

Neuropsychological Evaluation: The Raven Standard Progressive Matrices Test indicated potential issues in reasoning and problem-solving, while the Verbal Fluency Test suggested difficulties with cognitive flexibility and memory, and the Trail Making Test revealed problems with attention and sequencing. Imaging Findings: The initial CT scan demonstrated displaced linear fractures in the right temporal bone and two brain contusions shortly after the incident. On his visit nine months later, SPECT imaging showed relative hypoperfusion in the right posterior parietal cortex and bilateral temporal lobes. An EEG revealed slow wave anomalies in the right temporooccipital area and sharp spasms in the left temporal region. One year later, a follow-up PET scan revealed diffuse hypometabolism in the left frontal lobe, parietal lobes, and cerebellum.

**Image:**

**Figure:**
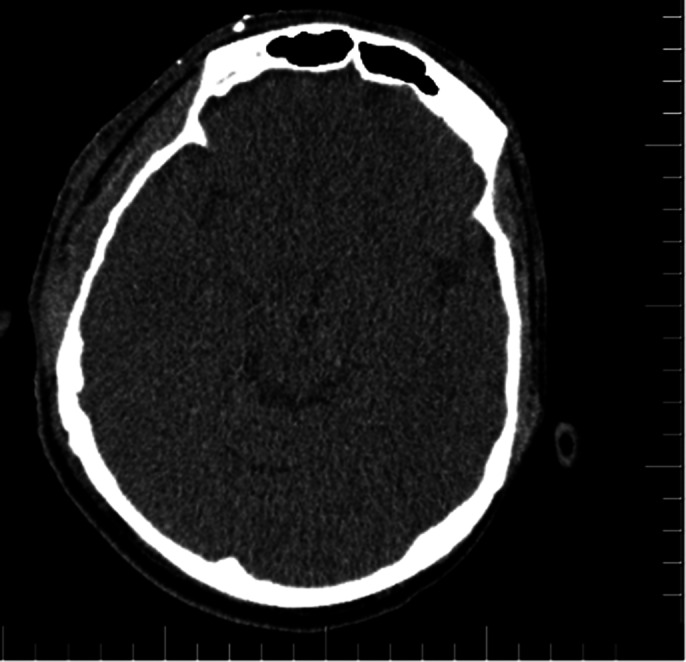

**Image 2:**

**Figure:**
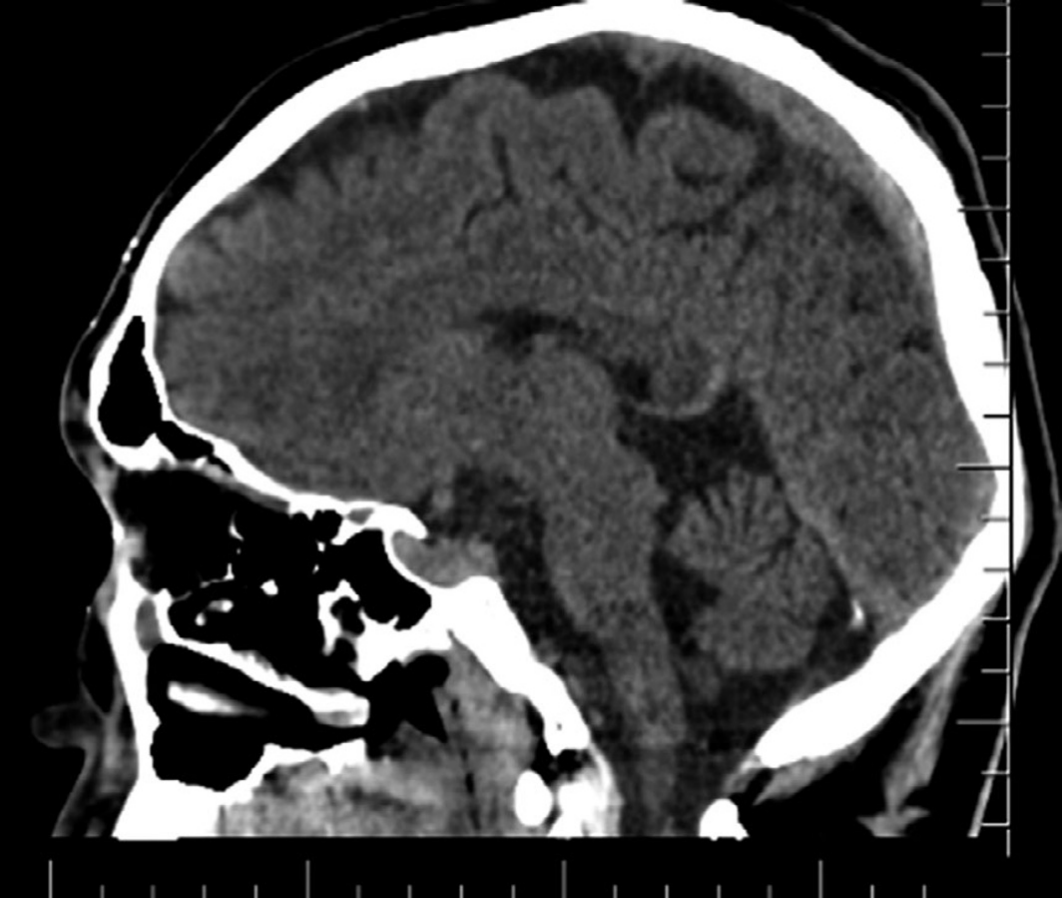

**Image 3:**

**Figure:**
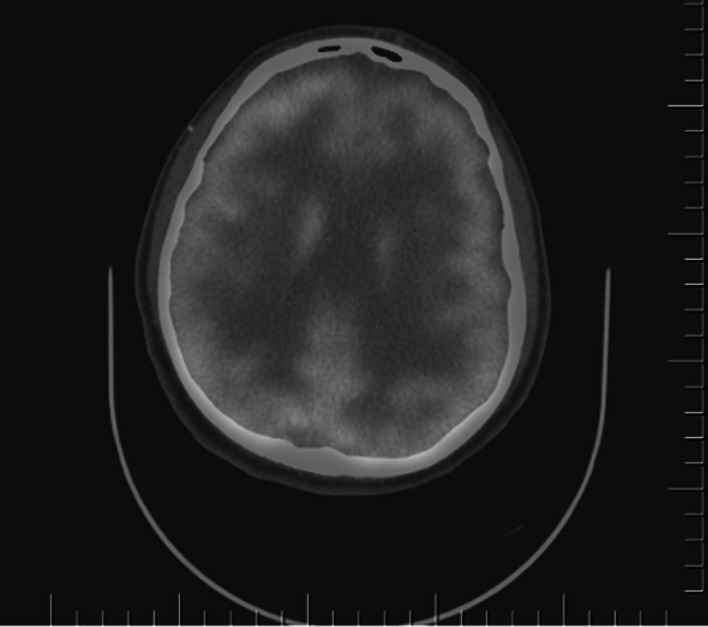

**Conclusions:**

This case emphasizes the role of follow-up neuroimaging in managing complex cases of combat-related TBI and PTSD. The progression from structural damage to later functional changes like hypometabolism in the frontal and parietal lobes illustrates how neuroimaging helps track the long-term impact of brain injuries and provides a comprehensive understanding of the evolving neurocognitive challenges. Continued neuroimaging is crucial for monitoring neurodegenerative processes and guiding adjustments in treatment (Koerte et al., 2015b). This approach supports a more targeted treatment plan, improving the veteran’s long-term prognosis (Wilde et al., 2015). Future studies should prioritize large, multi-site longitudinal research to track long-term neurotrauma effects through imaging, providing insights into neurodegeneration, recovery, and neuroplasticity.

**Disclosure of Interest:**

None Declared

